# Effects of Gene–Lifestyle Interaction on Obesity Among Students

**DOI:** 10.3390/genes15121506

**Published:** 2024-11-24

**Authors:** Emiliya S. Egorova, Kamilla K. Aseyan, Elvina R. Bikbova, Anastasia E. Zhilina, Elena V. Valeeva, Ildus I. Ahmetov

**Affiliations:** 1 Laboratory of Genetics of Aging and Longevity, Kazan State Medical University, 420012 Kazan, Russia; jastspring@yandex.ru (E.S.E.); aseyan.kamilla@yandex.ru (K.K.A.); ytii4686@gmail.com (E.R.B.); zhilina_anastasia01@mail.ru (A.E.Z.);; 2 Research Institute for Sport and Exercise Sciences, Liverpool John Moores University, Liverpool L3 5AF, UK

**Keywords:** lifestyle genetics, DNA, polymorphism, genotype, GxE, gene–lifestyle interaction, obesity, nutrition, eating behavior

## Abstract

Background: Obesity is a global health issue influenced primarily by genetic variants and environmental factors. This study aimed to examine the relationship between genetic and lifestyle factors and their interaction with obesity risk among university students. Methods: A total of 658 students from the same university participated in this study, including 531 females (mean age (SD): 21.6 (3.9) years) and 127 males (21.9 (4.6) years). Among them, 550 were classified as normal weight or underweight (456 females and 94 males), while 108 were identified as overweight or obese (75 females and 33 males). All the participants underwent anthropometric and genetic screening and completed lifestyle and sleep quality questionnaires. Results: The polygenic risk score, based on seven genetic variants (*ADCY3* rs11676272, *CLOCK* rs1801260, *GPR61* rs41279738, *FTO* rs1421085, *RP11-775H9.2* rs1296328, *SLC22A3* rs9364554, and *TFAP2B* rs734597), explained 8.3% (*p* < 0.0001) of the variance in body mass index (BMI). On the other hand, lifestyle factors—such as meal frequency, frequency of overeating, nut consumption as a snack, eating without hunger, frequency of antibiotic use in the past year, symptoms of dysbiosis, years of physical activity, sleep duration, bedtime, ground coffee consumption frequency, and evening coffee consumption time—accounted for 7.8% (*p* < 0.0001) of the variance in BMI. The model based on gene–environment interactions contributed 15% (*p* < 0.0001) to BMI variance. Conclusions: This study revealed that individuals with a higher genetic predisposition, as defined by the seven polymorphic loci, are more susceptible to becoming overweight or obese under certain lifestyle conditions.

## 1. Introduction

The World Health Organization (WHO) defines obesity as a condition characterized by an excessive accumulation of adipose tissue, which adversely impacts human health [1]. It is estimated that approximately 2 billion adults aged 18 years and older worldwide are overweight, with 650 million classified as obese [2,3]. Overweight and obesity are among the leading risk factors for mortality, contributing to at least 2.8 million deaths annually [1]. Notably, the association between body mass index (BMI) and mortality is stronger in younger age groups [4,5]. Epidemiological studies have shown that these conditions not only reduce life expectancy but also increase the prevalence of functional limitations [6].

The rising prevalence of obesity is primarily due to lifestyle changes in an obesogenic environment, characterized by reduced physical activity and increased consumption of high-calorie foods. Additionally, substantial evidence supports the role of genetic factors in body weight regulation [7,8,9]. However, recent genome-wide association studies (GWAS) indicate that identified candidate genes account for only about 6% of the variability in BMI [10]. This limited contribution underscores the complex interplay between genetic variants and environmental factors [11].

The conventional approach to investigating gene–environment–lifestyle interactions in obesity has been to examine the impact of genes (exposure) on obesity (outcome) in groups stratified by an environmental factor (e.g., physically active vs. inactive individuals). This approach allows for examining how environmental factors modify the association between exposure and outcome [12]. Gene-by-environment (GxE) interaction refers to how a person’s genetic makeup interacts with environmental factors to influence their phenotype, meaning the effect of genes can vary depending on the environmental context [13].

The concept of gene-by-environment interaction in obesity can be defined as the influence of a genetic variant on BMI, which may vary across different environmental conditions. Recent studies have demonstrated that genetic predisposition to obesity can be influenced by various lifestyle factors, including alcohol consumption, sweetened beverages, smoking, diet, and physical activity. The impact of physical activity levels on obesity has been confirmed in multiple studies [14,15,16,17]. Additionally, the role of television viewing habits in obesity has been examined [18], as well as the relationship between sleep quality and obesity [19].

While many other factors that appear to contribute to the risk of obesity have not been studied in the context of gene–environment interactions, such factors include human behaviors related to circadian rhythms (e.g., bedtime and coffee consumption patterns), as well as antibiotic use and intestinal dysbiosis. It is therefore evident that further investigation and replication of previous findings in diverse populations are required to gain a deeper understanding of the influence of environmental factors on genetic predisposition to obesity. Continued research on gene–environment interactions is essential to enhance our comprehension of the etiology of obesity and to facilitate the development of personalized prevention and treatment strategies.

The aim of this study was to examine the relationship between genetic and lifestyle factors, as well as their interaction with obesity risk, in a homogeneous cohort of students attending the same university and therefore living under partly identical environmental conditions.

## 2. Materials and Methods

### 2.1. Ethical Approval

This research was conducted in accordance with the ethical standards established by the Declaration of Helsinki of the World Medical Association and received approval from the Local Ethical Committee of Kazan State Medical University (protocol #10, dated 21 November 2023). Prior to participation, all the students were thoroughly informed about this study’s purpose and procedures, and each provided written consent to participate voluntarily.

### 2.2. Participants

The study group comprised university students. This study included 658 students: 338 of Tatar ethnicity, 225 of Russian ethnicity, and 95 of mixed origin. The mean age (SD) of the participants was 21.6 (4.0) years. The sample consisted of 531 females (age 21.6 (3.9) years) and 127 males (age 21.9 (4.6) years). Individuals engaged in professional sports, pregnant or lactating, or suffering from serious medical conditions (e.g., cancer and cerebral palsy) were excluded from the study. Additionally, individuals unable to engage in physical activity due to underlying health conditions were also excluded. This study included a total of 658 participants who met the eligibility criteria and provided informed consent. Age was recorded directly from the participants during the bioimpedance analysis, and ethnicity information was collected via a questionnaire.

### 2.3. Anthropometric Measurement

All the participants underwent anthropometric measurements to assess overweight and obesity status. Body weight and composition were measured using bioelectrical impedance (Tanita MC-780 MAN, Tanita, Japan). The parameters collected included total body weight (kg), fat-free mass (FFM, kg), fat mass (FM, kg), and body mass index (BMI, kg/m^2^). The measurements were taken in a fasting state.

### 2.4. Lifestyle Variables

The participants completed a lifestyle questionnaire. To identify new factors associated with obesity risk, we developed a customized questionnaire that included questions about daily meal patterns, such as meal frequency and timing (e.g., the timing of the last meal), frequency of overeating, and instances of eating without hunger. They also reported the number of daily snacks and the frequency and timing of beverages consumed, including coffee, caffeinated drinks, decaffeinated coffee, green tea, and black tea. The questionnaire assessed the frequency of consumption of various food items such as white meat, red meat, fish, dairy products, vegetables, fruits, juices, fast food, sausages, processed foods, and confectionery, as well as daily water intake.

Additionally, the participants provided details on their outdoor activities, including the frequency, duration, and intensity of physical exercise. To assess sleep quality, the participants completed the Pittsburgh Sleep Quality Index (PSQI) [20], which consists of 19 questions covering aspects such as typical bedtimes, wake-up times, time taken to fall asleep, and average hours of sleep per night.

### 2.5. SNP Selection and Genotyping

A total of 12 genetic variants were selected for inclusion in this study based on their consistent association with obesity traits as reported in previous studies [21,22,23]. The selected genetic variants included *ADCY3* rs11676272, *CLOCK* rs1801260, *GPR61* rs41279738, *FTO* rs14210, *RP11-775H9.2* rs1296328, *SLC22A3* rs9364554, and *TFAP2B* rs734597. These gene polymorphisms were also associated with various circadian rhythm traits, such as sleep duration, daytime sleepiness, and chronotype, based on summary statistics from the UK Biobank (https://genetics.opentargets.org; accessed on 15 October 2024).

Buccal epithelium samples, collected with disposable sterile probes, were used for a molecular genetic analysis. DNA was extracted using the sorbent method and the DNA-sorb-AM reagent kit (FBUN Central Research Institute of Epidemiology of Rospotrebnadzor, Moscow, Russia), following the manufacturer’s instructions. Gene polymorphisms were identified via real-time polymerase chain reaction using Testgen kits (Ulyanovsk, Russia).

### 2.6. Genetic Risk Score Calculation

Each genetic polymorphism was weighted based on its relative effect size (coefficient β) obtained from a regression analysis of BMI and the polymorphic locus under study. The weighted score of the genetic variants was calculated according to previously described methods [14,18,24,25,26]. The genotypes were assigned values of 0, 1, or 2 based on the number of risk alleles, which were then weighted by multiplying by the β coefficient. Given the additive genetic architecture of the BMI, the weighted scores for the seven gene variants were summed to estimate the cumulative effect of these gene polymorphisms on BMI. The polygenic risk score for the seven gene variants was calculated using the following formula:

G_BMI_ = β_1_SNP_1_ + … β_7_SNP_7_,

where G—genetic risk and β—effect size of genotype.

### 2.7. Lifestyle Risk Score Calculation

The weighted environmental factors were calculated using a similar approach. A total of 11 lifestyle parameters associated with BMI were included to evaluate the influence of environmental factors on BMI. Additionally, lifestyle parameters that increased the model’s coefficient of determination were incorporated. To incorporate categorical variables into the regression analysis, a matrix of dummy variables was constructed. Each environmental factor was assigned a value of 1 if it contributed to the risk of overweight or obesity, or 0 if it did not. A weighted assessment of the environmental factors was conducted using the β coefficient.

### 2.8. Statistical Analysis

Statistical analyses were performed using GraphPad InStat Version 3.05 (GraphPad Software, Inc., San Diego, CA, USA) software. The differences in allele and genotype frequencies between the samples and Hardy–Weinberg equilibrium compliance were tested using the χ^2^ test. A *p* value < 0.05 was considered statistically significant. Multiple regression analysis was used to assess the relationship between BMI and lifestyle parameters, including covariates such as sex, age, ethnicity, and physical activity level. The Benjamini–Hochberg correction was applied to control the false discovery rate (FDR) for multiple testing. The parametric data were checked for Gaussian distribution using the Kolmogorov–Smirnov test, with a *p* value < 0.05 indicating non-normality. For data not meeting the Gaussian distribution criteria, a Box–Cox transformation was applied, followed by a normality check.

To evaluate the combined effect of genetic variants (G), environmental factors (E), and gene–environment interactions (G × E) on BMI, a multiple regression analysis was conducted. The dependent variable was BMI, and the independent variables included sex, age, ethnicity, weighted genotype scores of seven polymorphic loci, and lifestyle parameters. The coefficient of determination (R^2^) was calculated to estimate the model’s influence on BMI. Additional covariates, such as age, sex, and ethnicity, were included in the BMI model. A linear regression model was constructed to assess the combined effect of genetic and environmental factors on BMI, adjusted for sex, age, and ethnicity.

## 3. Results

### 3.1. Anthropometric Characteristics

Among the 658 participants, 550 students (456 females and 94 males; mean age (SD): 21.4 (3.6) years) were classified as normal weight or underweight, while 108 students (75 females and 33 males; mean age: 22.9 (5.6) years) were classified as overweight or obese. Males classified as underweight or normal weight (i.e., non-obese) were significantly younger than males categorized as overweight or obese (*p* = 0.0002). No age differences were observed between the two subgroups of females. Both non-obese males and females had significantly (*p* < 0.0001) lower BMI, fat mass, and fat-free mass compared to overweight or obese individuals (Table 1).

### 3.2. Association Between Genetic Variants and BMI

**Figure 1:**
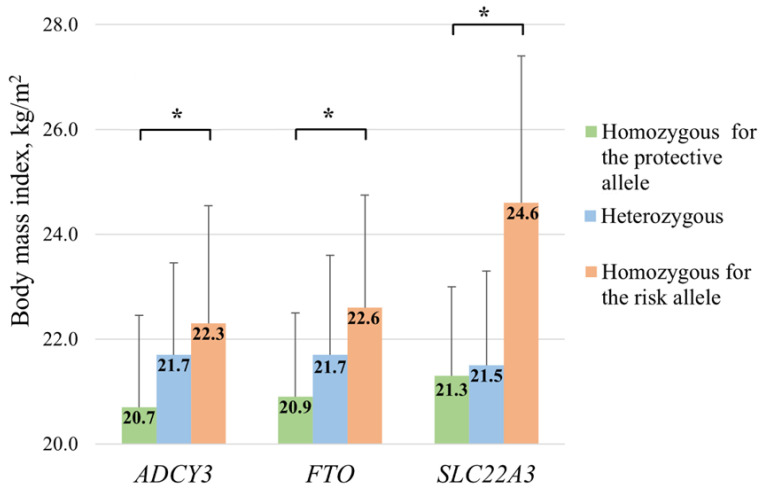
The mean BMI among individuals with different genotypes of the *ADCY3* rs11676272, FTO *rs1421085*, and *SLC22A3* rs9364554 polymorphisms. Protective alleles: *ADCY3* A, *FTO* T, and *SLC22A3* C. Risk alleles: *ADCY3* G, *FTO* C, and *SLC22A3* T. * *p* < 0.05.

The genotype distributions for the *ADCY3* rs11676272, *CLOCK* rs1801260, *FTO* rs1421085, *GPR61* rs41279738, *RP11-775H9.2* rs1296328, *SLC22A3* rs9364554, and *TFAP2B* rs734597 polymorphisms were in Hardy–Weinberg equilibrium (*p* > 0.05). To identify genetic variants associated with obesity, a case-control study was conducted, comparing allelic frequencies between overweight or obese (cases) and non-obese (controls) participants. When allelic frequencies differed by ethnicity (Supplementary Table S1), separate analyses were performed. After correcting for multiple testing, we identified associations between three genetic variants and BMI that aligned with findings in the literature, where obesity-related risk alleles were more prevalent among overweight or obese subjects. Specifically, the frequencies of the *ADCY3* rs11676272 G (60.3 vs. 41.0%, *p* = 0.03), *FTO* rs1421085 C (46.7 vs. 32.0%, *p* = 0.04), and *SLC22A3* rs9364554 T (38.0 vs. 23.7%, *p* = 0.01) alleles were significantly higher in overweight or obese individuals compared to non-obese participants (Supplementary Table S2). Furthermore, BMI (SD) significantly (*p* < 0.05) increased with an increase in the number of risk alleles in the whole cohort (*ADCY3*: 20.7 (3.5) → 21.7 (3.2) → 22.3 (3.4) kg/m^2^; *FTO*: 20.9 (3.5) → 21.7 (3.8) → 22.6 (3.6) kg/m^2^; and *SLC22A3*: 21.3 (4.5) → 21.5 (4.3) → 24.6 (5.6) kg/m^2^) (Figure 1).

Although the remaining four SNPs (*CLOCK* rs1801260, *GPR61* rs41279738, *RP11-775H9.2* rs1296328, and *TFAP2B* rs734597) did not show significant associations with BMI, we considered it justified to include all seven SNPs in the polygenic analysis, as each had been previously identified through genome-wide association studies—a common approach to minimize false-negative results. Consequently, a weighted polygenic risk score (Supplementary Table S3) based on these seven gene variants (*ADCY3* rs11676272, *CLOCK* rs1801260, *GPR61* rs41279738, *FTO* rs1421085, *RP11-775H9.2* rs1296328, *SLC22A3* rs9364554, and *TFAP2B* rs734597) explained 8.3% (*p* < 0.0001) of the variance in body mass index (BMI).

### 3.3. Association Between Lifestyle Parameters and BMI

**Figure 2:**
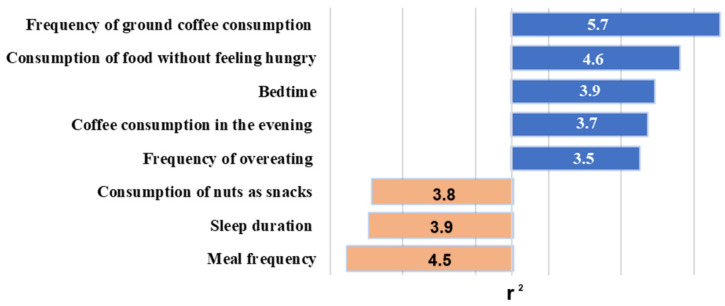
Coefficients of determination (r^2^) for lifestyle factors associated with BMI: blue indicates positive relationships, while orange indicates negative relationships.

A multiple regression analysis was conducted to examine the relationship between BMI and various lifestyle parameters of the subjects, adjusted for sex, age, ethnicity, and physical activity level. This analysis identified eight lifestyle factors significantly associated with BMI (Figure 2).

Data from the lifestyle questionnaires revealed several positive associations with BMI: frequency of overeating (r^2^ = 3.5; β = 0.006; and *p* = 0.03), consumption of food without feeling hungry (r^2^ = 4.6; β = 0.006; and *p* = 0.003), frequency of ground coffee consumption (r^2^ = 5.7; β = 0.007; and *p* = 0.0004), and evening coffee consumption (r^2^ = 3.7; β = 0.003; and *p* = 0.02). Additionally, individuals who consumed fewer meals had a higher BMI (r^2^ = 4.5; β = −0.004; and *p* = 0.003), while those who preferred nuts as snacks exhibited lower BMI values (r^2^ = 3.8; β = −0.0014; and *p* = 0.02). The analysis of the Pittsburgh Sleep Quality Questionnaire indicated that shorter sleep duration was associated with a greater BMI (r^2^ = 3.9; β = −0.008; and *p* = 0.01) among men (n = 92), and later bedtime was associated with higher BMI values (r^2^ = 3.9; β = 0.003; and *p* = 0.009).

**Figure 3:**
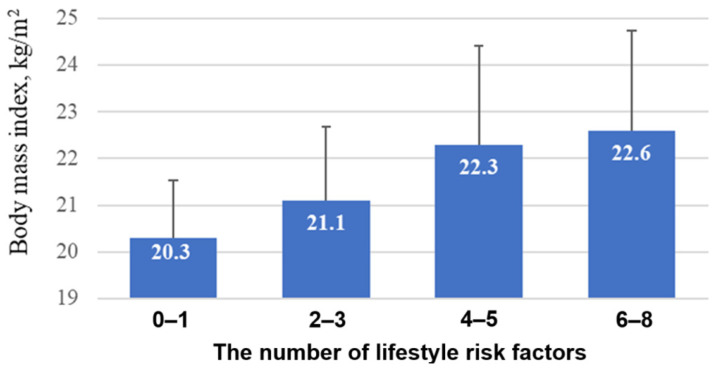
Relationship between the number of lifestyle risk factors and BMI. *p* < 0.0001 for the linear trend.

Additionally, we investigated the correlation between body mass index (BMI) and the number of risky lifestyle parameters. As shown in Figure 3, the presence of two additional lifestyle risk factors was significantly associated with an increase in BMI (20.3 (2.5) → 21.1 (3.2) → 22.3 (4.2) → 22.6 (4.3) kg/m^2^; *p* < 0.0001 for the linear trend). When the number of lifestyle factors ranged from six to eight, BMI reached its peak value of 22.6 kg/m^2^.

Next, we developed a model that accounted for all these lifestyle risk factors. To enhance the model’s robustness, additional factors—including years of physical activity, antibiotic intake over the past year, and frequency of dysbiosis symptoms—were included (Supplementary Table S4). Overall, these factors accounted for 7.8% of the variance in BMI (*p* < 0.0001).

### 3.4. Gene–Lifestyle Interaction

To evaluate the combined influence of lifestyle and genetic factors on the risk of overweight and obesity, the following multiple regression model was constructed, incorporating gene–lifestyle interactions:

Y = 1.5 + 0.001C_1_ − 0.01C_2_ + 0.003C_3_ + 68.4G × E,

where Y is BMI, C_1_ is ethnicity, C_2_ is sex, C_3_ is age, and GxE is gene–lifestyle interaction.

**Figure 4:**
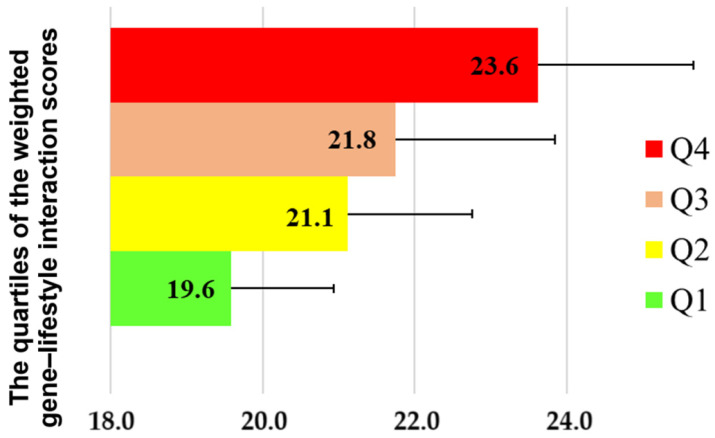
Distribution of BMI by quartile of weighted gene–lifestyle interaction score.

This model explained 15.0% of the variance in BMI among subjects (*p* < 0.0001). Additionally, the interaction between individual lifestyle factors and the genetic risk score was assessed. A significant interaction was identified between the polygenic score and several lifestyle variables, including meal frequency, frequency of overeating, nut consumption as a snack, years of physical activity, sleep duration, and bedtime (Supplementary Table S5). As shown in Figure 4, the quartile analysis of the weighted gene–lifestyle interaction score reveals a positive correlation between quartiles and BMI (*p* < 0.0001). Specifically, the mean (SD) BMI for individuals in the fourth quartile was 23.6 (4.1) kg/m^2^, which is 1.8 kg/m^2^ higher than that of individuals in the third quartile (21.8 (4.2) kg/m^2^), 2.5 kg/m^2^ higher than those in the second quartile (21.1 (3.3) kg/m^2^), and 4.0 kg/m^2^ higher than individuals in the first quartile (19.6 (2.7) kg/m^2^). This indicates that BMI increases with a higher weighted score, reflecting the combined genetic and environmental risk factors for obesity.

## 4. Discussion

Obesity results from a complex interplay of genetic and environmental influences. Over the past decade, significant efforts have been made to explore how these factors interact in the context of obesity. The objective of these studies is to elucidate the network of interactions involved in the development of complex diseases such as obesity, where multiple genes and environmental factors can modulate individual risk [27]. Traditional methodologies for examining gene–environment interactions in obesity typically assess the impact of genetic predisposition on the development of the condition, with environmental factors acting as moderators of the relationship between genetic influence and BMI [12].

In this study, we evaluated three models to assess the impacts of genetic, environmental, and gene–environment interactions on BMI variability among students. The first model included genetic variants known to be associated with obesity—specifically, *ADCY3* rs11676272, *CLOCK* rs1801260, *GPR61* rs41279738, *FTO* rs1421085, *RP11-775H9.2* rs1296328, *SLC22A3* rs9364554, and *TFAP2B* rs734597—and explained 8.3% of the variance in BMI. These genes are critical for regulating energy homeostasis and circadian rhythms, and previous studies have associated these polymorphisms with obesity [21,22,23].

The second model focused on environmental factors, incorporating lifestyle parameters such as the frequency of overeating, consumption of food without feeling hungry, meal frequency, ground coffee consumption, timing of coffee consumption (evening versus morning), sleep duration, bedtime, consumption of nuts as a snack, years of physical activity, frequency of antibiotic use in the past year, and the presence of dysbiosis symptoms. The findings of this study align with the existing literature, which links a high frequency of overeating [28,29], frequent consumption of food without hunger [29], short sleep duration [30,31], late bedtimes [32,33], and low levels of physical activity [34,35] to an increased risk of obesity.

Notably, our study presents a novel finding: a high frequency of coffee consumption and evening coffee consumption are linked to an increased risk of overweight and obesity. Although evidence suggests that coffee consumption can have favorable effects on BMI and weight loss [36,37], recent studies indicate it may also negatively impact body composition [38,39,40]. A recent large-scale genome-wide association study (GWAS) identified genes associated with coffee intake, substance use, and obesity-related traits. Notably, these genes are predominantly expressed in the brain and appear to influence human behavior. This hypothesis is supported by findings from a recent randomized controlled trial, which demonstrated a significant link between coffee consumption and an increased desire for sweet foods, as well as higher fructose intake and triglyceride levels [41]. Furthermore, it was shown that individuals with elevated genetic risk (based on three genetic variants) experienced a 50% increase in insulin resistance when consuming high amounts of coffee (over 10 cups per day) [42]. This suggests that the consumption of coffee may stimulate additional intake of sweet or fatty foods, contributing to weight gain. Moreover, Costa et al. [43] conducted a two-year prospective study revealing that coffee consumption is linked to increased obesity, particularly abdominal obesity, as well as decreased muscle quality. Mendelian randomization analyses highlighted differing effects of coffee consumption and plasma caffeine levels on BMI [44]. The findings indicated that coffee consumption correlates with increased BMI, while higher caffeine levels are associated with a decrease in BMI. This multidirectional effect may arise from individuals with slow caffeine metabolism resulting in lower plasma levels—compensating by increasing their caffeine intake.

The timing of coffee consumption may also mediate its effects. In the present study, subjects with a high genetic predisposition exhibited a higher BMI when consuming coffee in the evening. Data from mouse studies suggest that caffeine consumed early in the active period inhibits weight gain associated with high-fat meals [45]. The lipolytic effects of morning coffee intake are attributed to increased expression of circadian rhythm genes that regulate metabolism and suppression of lipogenesis gene expression [45].

The third model, which estimated the impact of gene–lifestyle interaction on BMI, demonstrated the most significant contribution at 15%. This study’s findings indicated that individuals with a genetic predisposition (based on seven polymorphic loci) displayed a heightened risk of overweight and obesity when exposed to specific lifestyle parameters. Moreover, stratifying individuals into quartiles based on their weighted gene–lifestyle interaction scores revealed a statistically significant increase in mean BMI values. Subjects in the fourth quartile had a BMI 4.0 kg/m^2^ higher than those in the first quartile. These findings underscore the role of the interaction between identified genetic and lifestyle factors in elevating obesity risk. Additionally, the complex interplay between all lifestyle factors and genetic polymorphisms accounted for a greater proportion of BMI variability than any single lifestyle factor alone. This reinforces the importance of adhering to all aspects of a healthy lifestyle to minimize the risk of developing obesity.

Previous GWASs have demonstrated the association of the studied polymorphic loci *ADCY3* rs1167627272, *CLOCK* rs1801260, *FTO* rs1421085, *GPR61* rs41279738, *RP11-775H9.2* rs1296328, *SLC22A3* rs9364554, and *TFAP2B* rs734597 with obesity-related phenotypes [25,26,27]. Of these, three genetic variants (*ADCY3* rs11676272, *FTO* rs1421085, and *SLC22A3* rs9364554) were significantly associated with BMI individually in our study, as well as all seven loci in combination. The *ADCY3* gene encodes adenyl cyclase 3, which catalyzes the conversion of ATP to cAMP, a molecule involved in signaling pathways for glucagon-like peptide 1, ghrelin, orexins, α-melanocyte-stimulating hormone, and leptin [46,47]. The *SLC22A3* gene (also known as organic cation transporter 3, OCT3) mediates norepinephrine uptake in white adipose tissue, which activates β3-adrenergic receptors to increase cAMP and protein kinase A levels, thereby enhancing lipolysis through hormone-sensitive lipase [48,49]. In mice, *Slc22a3* promotes adipose browning, thermogenesis, and mitochondrial biogenesis [49]. The *FTO* gene encodes α-ketoglutarate-dependent dioxygenase, with many variants associated with obesity-related traits. Notably, previous research has identified an interaction between the FTO rs1421085 locus and various environmental factors. Specifically, studies indicated that dietary fiber intake [50], trans fat consumption [51], and adherence to the Mediterranean diet [52] influenced obesity risk among genetically predisposed individuals. Furthermore, factors such as fast food and sugary beverage consumption [53], meal frequency [54], and physical activity levels [55] have also been shown to affect obesity risk in this population.

Previous studies have shown that the interplay between genetic variants and factors such as physical activity level, smoking, alcohol consumption, and socioeconomic status contributes to BMI variability. Often, polygenic risk scores are calculated based on the polymorphic loci identified by Locke et al. [56]. For instance, a large-scale study found that genotype–smoking interactions accounted for 4.0% of BMI variability [57]. A recent British Biobank investigation involving over 4 million single-nucleotide polymorphisms reported a more modest contribution of environmental factors [58]. This study indicated that the MET score, the number of pack-years of smoking, and the frequency of alcohol consumption interacted with genetic variants to partially explain BMI variance, with heritability contributions of 0.45% for the MET score, 0.52% for pack-years of smoking, and 0.32% for alcohol frequency. Sulc et al. [59] reported a gene-mediated contribution of 1.9% to BMI variability using a model that treated environmental factors as random effects, requiring only outcome and genetic data. Additionally, interactions between polymorphic loci and age accounted for 0.4%, while neuroticism score, physical activity, and alcohol consumption frequency contributed 0.7%, 0.3%, and 0.3%, respectively, to BMI variability. In this study, we found no evidence that interactions between alcohol consumption and smoking with genetic variants contributed to BMI variance. Additionally, the student cohort was largely homogeneous regarding socioeconomic status, limiting our ability to explore the potential influences of socioeconomic factors and genotype on BMI variability.

It should be noted that our study has limitations due to its cross-sectional design and reliance on self-reported data. While self-reporting is common in epidemiological research, it can introduce biases, such as recall and social desirability bias. Nonetheless, some of the questionnaires used have been validated and shown to be reliable in previous studies, lending confidence to our findings despite the limitations inherent to self-reported data. We also acknowledge that this study used a limited number of genetic markers associated with obesity, despite the existence of several hundred known obesity-related DNA polymorphisms [10,56]. In addition, numerous environmental factors influencing obesity were not considered in this study, which represents a limitation. Finally, polygenic risk scores (PRSs) have several limitations, including limited predictive accuracy for complex traits, poor generalizability across different ancestries, inability to account for environmental factors, difficulty in interpreting individual results, and a lack of robust clinical applications due to the relatively small effect sizes of individual genetic variants [60].

## 5. Conclusions

This study revealed that individuals with a higher genetic predisposition, as defined by the seven polymorphic loci, are more susceptible to becoming overweight or obese under certain lifestyle conditions. The gene–lifestyle interaction model we developed accounted for 15% of the variance in BMI. This model incorporated genetic variants that have been underexplored in similar studies, alongside a broad range of environmental factors encompassing various aspects of an individual’s life (dietary habits, physical activity, circadian rhythms, antibiotic intake, etc.). In contrast, many prior studies have focused on a narrower set of lifestyle factors. Our findings underscore that substantial contributions to BMI variance arise from the complex interplay of genetic and environmental factors rather than from any single factor. These insights may inform the development of prevention strategies and non-pharmacological obesity management approaches that integrate genetic data with individual environmental contexts, potentially enhancing the effectiveness of existing interventions [61,62,63].

## Figures and Tables

**Table 1:** Anthropometric characteristics of participants.

Traits	Males		Females	
	Non-Obese (*n* = 94)	Overweight or Obese (*n* = 33)	Non-Obese (*n* = 456)	Overweight or Obese (*n* = 75)
Age, years	21.5 (3.7)	24.9 (6.9) **	20.8 (2.8)	22.0 (4.7)
BMI, kg/m^2^	21.0 (2.0)	28.3 (2.7) *	20.0 (2.0)	28.1 (2.8) *
Fat mass, kg	9.9 (4.6)	22.2 (5.7) *	14.1 (4.2)	26.8 (5.5) *
Fat-free mass, kg,	61.6 (7.3)	66.3 (6.5) *	30.1 (5.3)	49.5 (4.8) *

* *p* < 0.0001 and ** *p* = 0.0002; data are means (SD).

## Data Availability

The data that support the findings of this study are available from the corresponding author upon reasonable request due to the privacy of the students.

## Supplementary Materials

The following supporting information can be downloaded at https://www.mdpi.com/article/10.3390/genes15121506/s1: Table S1: Differences in genotype distribution and allele frequencies across ethnicities. Table S2: Genotype and allele frequencies of significant gene polymorphisms by ethnicity and BMI group. Table S3: Calculation of weighted polygenic risk score for obesity. Table S4: Binary values assigned to risk factors based on questionnaire responses. Table S5: Contribution of genetic risk score and lifestyle factor interactions to BMI variance.

## References

[1] WHO Information Bulletins Obesity and Overweight. https://www.who.int/ru/news-room/fact-sheets/detail/obesity-and-overweight.

[2] Yanovski J.A. (2018). Obesity: Trends in underweight and obesity—Scale of the problem. Nat. Rev. Endocrinol..

[3] GBD 2017 Diet Collaborators (2019). Health effects of dietary risks in 195 countries, 1990–2017: A systematic analysis for the Global Burden of Disease Study 2017. Lancet.

[4] Bhaskaran K., Dos-Santos-Silva I., Leon D.A., Douglas I.J., Smeeth L. (2018). Association of BMI with overall and cause-specific mortality: A population-based cohort study of 3.6 million adults in the UK. Lancet Diabetes Endocrinol..

[5] Di Angelantonio E., Bhupathiraju S.N., Wormser D., Gao P., Kaptoge S., Berrington de Gonzalez A., Cairns B.J., Huxley R., Jackson C.H., Global BMI Mortality Collaboration (2016). Body-mass index and all-cause mortality: Individual-participant-data meta-analysis of 239 prospective studies in four continents. Lancet.

[6] Townsend T.N., Mehta N.K. (2020). Contributions of obesity and cigarette smoking to incident disability: A longitudinal analysis. Prev. Med..

[7] Silventoinen K., Li W., Jelenkovic A., Sund R., Yokoyama Y., Aaltonen S., Piirtola M., Sugawara M., Tanaka M., Matsumoto S. (2022). Changing genetic architecture of body mass index from infancy to early adulthood: An individual based pooled analysis of 25 twin cohorts. Int. J. Obes..

[8] Silventoinen K., Jelenkovic A., Sund R., Latvala A., Honda C., Inui F., Tomizawa R., Watanabe M., Sakai N., Rebato E. (2020). Genetic and environmental variation in educational attainment: An individual-based analysis of 28 twin cohorts. Sci. Rep..

[9] Bouchard C. (2021). Genetics of Obesity: What We Have Learned Over Decades of Research. Obesity.

[10] Yengo L., Sidorenko J., Kemper K.E., Zheng Z., Wood A.R., Weedon M.N., Frayling T.M., Hirschhorn J.N., Yang J., Visscher P.M. (2018). Meta-analysis of genome-wide association studies for height and body mass index in ∼700,000 individuals of European ancestry. Hum. Mol. Genet..

[11] Kim M.S., Shim I., Fahed A.C., Do R., Park W.-Y., Natarajan P., Khera A.V., Won H.-H. (2024). Association of genetic risk, lifestyle, and their interaction with obesity and obesity-related morbidities. Cell Metab..

[12] Pérusse L., Jacob R., Drapeau V., Llewellyn C., Arsenault B.J., Bureau A., Labonté M.È., Tremblay A., Vohl M.C. (2022). Understanding Gene-Lifestyle Interaction in Obesity: The Role of Mediation versus Moderation. Lifestyle Genom..

[13] Hunter D.J. (2005). Gene-environment interactions in human diseases. Nat. Rev. Genet..

[14] Rask-Andersen M., Karlsson T., Ek W.E., Johansson A. (2017). Gene-environment interaction study for BMI reveals interactions between genetic factors and physical activity, alcohol consumption, and socioeconomic status. PLoS Genet..

[15] Chermon D., Birk R. (2024). Gene-Environment Interactions Significantly Alter the Obesity Risk of SH2B1 rs7498665 Carriers. J. Obes. Metab. Syndr..

[16] Li S., Zhao J.H., Luan J., Ekelund U., Luben R.N., Khaw K.T., Wareham N.J., Loos R.J. (2010). Physical activity attenuates the genetic predisposition to obesity in 20,000 men and women from the EPIC-Norfolk prospective population study. PLoS Med..

[17] Xue H., Zhang X., Li D., Chen M., Luo J., Gong Y., Lv X., Quan L., He F., Zhang L. (2021). Relevance of Physical Activities, Sedentary Behaviors, and Genetic Predisposition in Body Fatness: Population-Based Study on Chinese Adults. Obes. Facts.

[18] Qi Q., Li Y., Chomistek A.K., Kang J.H., Curhan G.C., Pasquale L.R., Willett W.C., Rimm E.B., Hu F.B., Qi L. (2012). Television watching, leisure time physical activity, and the genetic predisposition in relation to body mass index in women and men. Circulation.

[19] Celis-Morales C., Lyall D.M., Guo Y., Steell L., Llanas D., Ward J., Mackay D.F., Biello S.M., Bailey M.E., Pell J.P. (2017). Sleep characteristics modify the association of genetic predisposition with obesity and anthropometric measurements in 119,679 UK Biobank participants. Am. J. Clin. Nutr..

[20] Buysse D.J., Reynolds C.F., Monk T.H., Berman S.R., Kupfer D.J. (1989). The Pittsburgh Sleep Quality Index: A new instrument for psychiatric practice and research. Psychiatry Res..

[21] Barton A.R., Sherman M.A., Mukamel R.E., Loh P.R. (2021). Whole-exome imputation within UK Biobank powers rare coding variant association and fine-mapping analyses. Nat. Genet..

[22] Hübel C., Gaspar H.A., Coleman J.R.I., Finucane H., Purves K.L., Hanscombe K.B., Prokopenko I., Graff M., Ngwa J.S., MAGIC investigators (2019). Genomics of body fat percentage may contribute to sex bias in anorexia nervosa. Am. J. Med. Genet. B Neuropsychiatr. Genet..

[23] Martin S., Cule M., Basty N., Tyrrell J., Beaumont R.N., Wood A.R., Frayling T.M., Sorokin E., Whitcher B., Liu Y. (2021). Genetic Evidence for Different Adiposity Phenotypes and Their Opposing Influences on Ectopic Fat and Risk of Cardiometabolic Disease. Diabetes.

[24] Keller M.C. (2014). Gene × environment interaction studies have not properly controlled for potential confounders: The problem and the (simple) solution. Biol. Psychiatry.

[25] Nakamura S., Fang X., Saito Y., Narimatsu H., Ota A., Ikezaki H., Shimanoe C., Tanaka K., Kubo Y., Tsukamoto M. (2023). Effects of gene-lifestyle interactions on obesity based on a multi-locus risk score: A cross-sectional analysis. PLoS ONE.

[26] Choi S.W., Mak T.S., O’Reilly P.F. (2020). Tutorial: A guide to performing polygenic risk score analyses. Nat. Protoc..

[27] Cano-Gamez E., Trynka G. (2020). From GWAS to Function: Using Functional Genomics to Identify the Mechanisms Underlying Complex Diseases. Front. Genet..

[28] Vasileiou V., Abbott S. (2023). Emotional eating among adults with healthy weight, overweight, and obesity: A systematic review and meta-analysis. J. Hum. Nutr. Diet..

[29] Teodoro M.C., Conceição E.M., de Lourdes M., Alves J.R., Neufeld C.B. (2021). Grazing’s frequency and associations with obesity, psychopathology, and loss of control eating in clinical and community contexts: A systematic review. Appetite.

[30] Larsen S.C., Horgan G., Mikkelsen M.K., Palmeira A.L., Scott S., Duarte C., Santos I., Encantado J., Driscoll R.O., Turicchi J. (2020). Association between objectively measured sleep duration, adiposity and weight loss history. Int. J. Obes..

[31] Bacaro V., Ballesio A., Cerolini S., Vacca M., Poggiogalle E., Donini L.M., Lucidi F., Lombardo C. (2020). Sleep duration and obesity in adulthood: An updated systematic review and meta-analysis. Obes. Res. Clin. Pract..

[32] Longo-Silva G., Pedrosa A.K.P., de Oliveira P.M.B., da Silva J.R., de Menezes R.C.E., Marinho P.d.M., Bernardes R.S. (2023). Beyond sleep duration: Sleep timing is associated with BMI among Brazilian adults. Sleep Med. X.

[33] Tse L.A., Wang C., Rangarajan S., Liu Z., Teo K., Yusufali A., Avezum Á., Wielgosz A., Rosengren A., Kruger I.M. (2021). Timing and Length of Nocturnal Sleep and Daytime Napping and Associations With Obesity Types in High-, Middle-, and Low-Income Countries. JAMA Netw. Open.

[34] Tammelin T., Laitinen J., Näyhä S. (2004). Change in the level of physical activity from adolescence into adulthood and obesity at the age of 31 years. Int. J. Obes. Relat. Metab. Disord..

[35] Curran F., Davis M.E., Murphy K., Tersigni N., King A., Ngo N., O’Donoghue G. (2023). Correlates of physical activity and sedentary behavior in adults living with overweight and obesity: A systematic review. Obesity Rev..

[36] Wang T., Huang T., Kang J.H., Zheng Y., Jensen M.K., Wiggs J.L., Pasquale L.R., Fuchs C.S., Campos H., Rimm E.B. (2017). Habitual coffee consumption and genetic predisposition to obesity: Gene-diet interaction analyses in three US prospective studies. BMC Med..

[37] Icken D., Feller S., Engeli S., Mayr A., Müller A., Hilbert A., de Zwaan M. (2016). Caffeine intake is related to successful weight loss maintenance. Eur. J. Clin. Nutr..

[38] Kim J.H., Park Y.S. (2017). Light coffee consumption is protective against sarcopenia, but frequent coffee consumption is associated with obesity in Korean adults. Nutr. Res..

[39] Lee J., Kim H.Y., Kim J. (2017). Coffee Consumption and the Risk of Obesity in Korean Women. Nutrients.

[40] Nicolopoulos K., Mulugeta A., Zhou A., Hyppönen E. (2020). Association between habitual coffee consumption and multiple disease outcomes: A Mendelian randomisation phenome-wide association study in the UK Biobank. Clin. Nutr..

[41] Magaña-de la Vega L., Martínez-López E., Sanchez-Murguia T., Madrigal-Juárez A., Rodríguez-Reyes S.C., Aguilar-Vega I., Torres-Castillo N. (2024). Effect of coffee intake on appetite parameters in woman with overweight or obesity: A pilot crossover randomized trial. Endocrinol. Diabetes Nutr..

[42] Daily J.W., Liu M., Park S. (2019). High genetic risk scores of SLIT3, PLEKHA5 and PPP2R2C variants increased insulin resistance and interacted with coffee and caffeine consumption in middle-aged adults. Nutr. Metab. Cardiovasc. Dis..

[43] Costa M.S.D., Pontes K.S.D.S., Guedes M.R., Barreto Silva M.I., Klein M.R.S.T. (2023). Association of habitual coffee consumption with obesity, sarcopenia, bone mineral density and cardiovascular risk factors: A two-year follow-up study in kidney transplant recipients. Clin. Nutr..

[44] Woolf B., Cronjé H.T., Zagkos L., Larsson S.C., Gill D., Burgess S. (2024). Comparison of caffeine consumption behavior with plasma caffeine levels as exposure measures in drug-target Mendelian randomization. Am. J. Epidemiol..

[45] Haraguchi A., Yamazaki T., Ryan C., Ito K., Sato S., Tamura K., Sekiguchi M., Cao S., Shibata S. (2022). Caffeine suppresses high-fat diet-induced body weight gain in mice depending on feeding timing. J. Funct. Foods.

[46] Ludwig M.G., Seuwen K. (2002). Characterization of the human adenylyl cyclase gene family: cDNA, gene structure, and tissue distribution of the nine isoforms. J. Recept. Signal Transduct. Res..

[47] Xu T.R., Yang Y., Ward R., Gao L., Liu Y. (2013). Orexin receptors: Multi-functional therapeutic targets for sleeping disorders, eating disorders, drug addiction, cancers, and other physiological disorders. Cell. Signal..

[48] Duncan R.E., Ahmadian M., Jaworski K., Sarkadi-Nagy E., Sul H.S. (2007). Regulation of lipolysis in adipocytes. Annu. Rev. Nutr..

[49] Song W., Luo Q., Zhang Y., Zhou L., Liu Y., Ma Z., Guo J., Huang Y. (2019). Organic cation transporter 3 (Oct3) is a distinct catecholamines clearance route in adipocytes mediating the beiging of white adipose tissue. PLoS Biol..

[50] Hosseini-Esfahani F., Koochakpoor G., Daneshpour M.S., Mirmiran P., Sedaghati-Khayat B., Azizi F. (2017). The interaction of fat mass and obesity associated gene polymorphisms and dietary fiber intake in relation to obesity phenotypes. Sci. Rep..

[51] Koochakpour G., Esfandiar Z., Hosseini-Esfahani F., Mirmiran P., Daneshpour M.S., Sedaghati-Khayat B., Azizi F. (2019). Evaluating the interaction of common FTO genetic variants, added sugar, and trans-fatty acid intakes in altering obesity phenotypes. Nutr. Metab. Cardiovasc. Dis..

[52] Hosseini-Esfahani F., Koochakpoor G., Daneshpour M.S., Sedaghati-Khayat B., Mirmiran P., Azizi F. (2017). Mediterranean Dietary Pattern Adherence Modify the Association between FTO Genetic Variations and Obesity Phenotypes. Nutrients.

[53] Hosseini-Esfahani F., Koochakpoor G., Mirmiran P., Daneshpour M.S., Azizi F. (2019). Dietary patterns modify the association between fat mass and obesity-associated genetic variants and changes in obesity phenotypes. Br. J. Nutr..

[54] Jääskeläinen A., Schwab U., Kolehmainen M., Kaakinen M., Savolainen M.J., Froguel P., Cauchi S., Järvelin M.R., Laitinen J. (2013). Meal frequencies modify the effect of common genetic variants on body mass index in adolescents of the northern Finland birth cohort 1986. PLoS ONE.

[55] Reddon H., Gerstein H.C., Engert J.C., Mohan V., Bosch J., Desai D., Bailey S.D., Diaz R., Yusuf S., Anand S.S. (2016). Physical activity and genetic predisposition to obesity in a multiethnic longitudinal study. Sci. Rep..

[56] Locke A.E., Kahali B., Berndt S.I., Justice A.E., Pers T.H., Day F.R., Powell C., Vedantam S., Buchkovich M.L., Yang J. (2015). Genetic studies of body mass index yield new insights for obesity biology. Nature.

[57] Robinson M.R., English G., Moser G., Lloyd-Jones L.R., Triplett M.A., Zhu Z., Nolte I.M., van Vliet-Ostaptchouk J.V., Snieder H., LifeLines Cohort Study (2017). Genotype-covariate interaction effects and the heritability of adult body mass index. Nat. Genet..

[58] Jung H.U., Kim D.J., Baek E.J., Chung J.Y., Ha T.W., Kim H.-K., Kang J.-O., Lim J.E., Oh B. (2023). Gene-environment interaction explains a part of missing heritability in human body mass index. Commun. Biol..

[59] Sulc J., Mounier N., Günther F., Winkler T., Wood A.R., Frayling T.M., Heid I.M., Robinson M.R., Kutalik Z. (2020). Quantification of the overall contribution of gene-environment interaction for obesity-related traits. Nat. Commun..

[60] Xiang R., Kelemen M., Xu Y., Harris L.W., Parkinson H., Inouye M., Lambert S.A. (2024). Recent advances in polygenic scores: Translation, equitability, methods and FAIR tools. Genome Med..

[61] Leońska-Duniec A., Jastrzębski Z., Jażdżewska A., Moska W., Lulińska-Kuklik E., Sawczuk M., Gubaydullina S.I., Shakirova A.T., Cięszczyk P., Maszczyk A. (2018). Individual Responsiveness to Exercise-Induced Fat Loss and Improvement of Metabolic Profile in Young Women is Associated with Polymorphisms of Adrenergic Receptor Genes. J. Sport. Sci. Med..

[62] Leonska-Duniec A., Cięszczyk P., Ahmetov I.I., Barh D., Ahmetov I. (2019). Genes and individual responsiveness to exercise-induced fat loss. Sports, Exercise, and Nutritional Genomics: Current Status and Future Directions.

[63] Bojarczuk A., Egorova E.S., Dzitkowska-Zabielska M., Ahmetov I.I. (2024). Genetics of Exercise and Diet-Induced Fat Loss Efficiency: A Systematic Review. J. Sports Sci. Med..

