# Supplementary material for: Effects of Gene–Lifestyle Interaction on Obesity Among Students
Source: Genes (Basel). 2024 Nov 24;15(12):1506. doi: 10.3390/genes15121506 (PMC11675936; doi:10.3390/genes15121506)
Supplement: Supplementary file 1 [file genes-15-01506-s001.zip › genes-3320706-supplementary.pdf]

Supplementary file

Table S1. Differences in genotype distribution and allele frequencies across ethnicities

SNP	Subjects	Ethnicity	n	Genotypes			p_1	G allele	
				AA	AG	GG		%	p_2
ADCY3 rs11676272	Individuals with BMI ≥25 kg/m²	Tatars	41	13	23	5	0.03*	41.0	0.02*
		Russians	29	5	13	11		60.3	
		Tatars	41	13	23	5	0.43	41.0	0.27
		Mestizos	8	1	5	2		56.3	
		Russians	29	5	13	11	0.67	60.3	0.78
		Mestizos	8	1	5	2		56.3	
**SNP**	**Subjects**	**Ethnicity**	**n**	**Genotypes**			P_1	**C allele**	
				**TT**	**TC**	**CC**		**%**	P_2
FTO rs1421085	All individuals	Tatars	292	116	131	45	0.99	37.8	1.00
		Russians	197	79	87	31		37.8	
		Tatars	292	116	131	45	0.11	37.8	0.03*
		Mestizos	77	22	37	18		47.4	
		Russians	197	79	87	31	0.14	37.8	0.04*
		Mestizos	77	22	37	18		47.4	
**SNP**	**Subjects**	**Ethnicity**	**n**	**Genotypes**			P_1	**C allele**	
				**CC**	**CT**	**TT**		**%**	P_2
SLC22A3 rs9364554	All individuals	Tatars	320	177	119	24	0.06	73.9	0.02*
		Russians	212	136	68	8		80.2	
		Tatars	320	177	119	24	0.22	73.9	0.19
		Mestizos	84	51	31	2		79.2	
		Russians	212	136	68	8	0.64	80.2	0.82
		Mestizos	84	51	31	2		79.2	

* $p_1 < 0.05$, statistically significant differences in genotype distribution between different ethnicities. $p_2 < 0.05$, statistically significant differences in allelic frequencies between different ethnicities.

Table S2. Genotype and allele frequencies of significant gene polymorphisms by ethnicity and BMI group.

	Ethnic group	Group	n	Genotypes			G allele		X^2	$p_{H\text{-}W}$
				AA	AG	GG	%	p		
ADCY3 rs11676272	Russian	With BMI <25 kg/m²	155	59	65	31	41.0	0.03*	2.74	0.09
		With BMI ≥25 kg/m²	29	5	13	11	60.3		0.11	0.73
	**Ethnic group**	**Group**	**n**	**Genotypes**			**C allele**		X^2	$p_{H\text{-}W}$
				TT	TC	CC	%	p		
FTO rs1421085	Russian & Tatar	With BMI <25 kg/m²	414	174	180	60	36.2	0.051	1.44	0.22
		With BMI ≥25 kg/m²	75	21	38	16	46.7		0.02	0.87
		With BMI <18,5 kg/m²	97	46	40	11	32.0	0.04*	0.26	0.60
		With BMI ≥25 kg/m²	75	21	38	16	46.7		0.02	0.87
	**Ethnic group**	**Group**	**n**	**Genotypes**			**T allele**		X^2	$p_{H\text{-}W}$
				CC	CT	TT	%	p		
SLC22A3 rs9364554	Tatar	With BMI <25 kg/m²	266	154	98	14	23.7	0.01*	0.1	0.75
		With BMI ≥25 kg/m²	54	23	21	10	38.0		1.64	0.20

p, p value for differences in allelic frequencies.; $p_{H\text{-}W}$, p value for testing Hardy-Weinberg equilibrium.
* $p < 0.05$, statistically significant differences.

Table S3. Calculation of weighted polygenic risk score for obesity

Nearest Gene	SNP	Allele major/minor	Risk allele	β coefficient (SE)	*p*
ADCY3	rs11676272	A/G	G	0.003 (0.003)	0.32
FTO	rs1421085	T/C	C	0.007 (0.003)	0.1
CLOCK	rs1801260	T/C	T	0.002 (0.003)	0.57
GPR61	rs41279738	T/G	G	0.01 (0.008)	0.16
RP11-775H9.2	rs1296328	A/C	A	0.005 (0.003)	0.09
TFAP2B	rs734597	G/A	A	0.003 (0.003)	0.54
SLC22A3	rs9364554	C/T	T	0.004 (0.003)	0.27

P value indicates association between the SNP and BMI using linear regression model adjusted for sex, age, ethnicity and physical activity.

Table S4. Binary values assigned to risk factors based on questionnaire responses

Risk Factor	Description	Value
Overeating Frequency	High frequency of overeating	1
	Low frequency of overeating	0
Consumption Without Hunger	High frequency of consumption without feeling hungry	1
	Low frequency of consumption without feeling hungry	0
Meal Frequency	Low frequency of meals (1-3 meals per day)	1
	High frequency of meals (4-6 meals per day)	0
Ground Coffee Consumption	High frequency (3 cups per day or more)	1
	Low frequency (2 cups per day or less)	0
Coffee Consumption Time	Evening coffee consumption time	1
	Morning and afternoon coffee consumption	0
Night Sleep Duration	Less than 7 hours of night sleep	1
	7 hours or more of night sleep	0
Bedtime	Bedtime at 00:00 hours or later	1
	Bedtime before 00:00 hours	0
Snack Consumption	Consumption of nuts as a snack	0
	Consumption of other snacks	1
Physical Activity Duration	Less than 6 months of physical activity	1
	More than 6 months of physical activity	0
Antibiotic Use Frequency	Frequent use (3 times a year or more)	1
	Infrequent use (2 times a year or less)	0
Dysbiosis Symptoms Frequency	High frequency of dysbiosis symptoms	1
	Rare frequency or no symptoms	0

Table S5. Contribution of genetic risk score and lifestyle factor interactions to BMI variance

Lifestyle variable	n	R^2	*P*	β
Meal frequency	275	7.0%	0.0006	188.1 (50.8)
Frequency of overeating	275	4.2%	0.02	108.6 (47.7)
Consumption of nuts as a snack	292	6.3%	0.0009	409.4 (129.7)
Frequency of consumption of food without feeling hungry	275	2.4%	0.16	65.0 (103.0)
Frequency of using of antibiotics per last year	275	2.8%	0.10	57.4 (47.6)
Frequency of dysbiosis symptoms	275	2.9%	0.09	72.5 (55.3)
Years of physical activity	269	7.3%	0.005	170.2 (48.5)
Sleep duration	265	7.1%	0.0007	79.5 (22.5)
Bedtime	272	5.3%	0.0058	193.3 (62.7)
Frequency of the ground coffee consumption	241	2.4%	0.23	103.6 (178.7)
Evening coffee consumption time	240	2.4%	0.23	67.8 (84.1)
